# Expression of il-23/th17 pathway in a murine model of coxsackie virus b3-induced viral myocarditis

**DOI:** 10.1186/1743-422X-8-301

**Published:** 2011-06-14

**Authors:** Fan Yang, Wei-Feng Wu, Yu-Luan Yan, Yu Pang, Qing Kong, Yan-Lan Huang

**Affiliations:** 1 Department of Cardiology, the First Affiliated Hospital of Guangxi Medical University, Guangxi Cardiovascular Institute, Shuang-Yong Road 6, Nanning, China

**Keywords:** IL-23, Th17, murine, viral myocarditis

## Abstract

**Background:**

The IL-23/Th17 pathway is implicated in the pathogenesis of a number of chronic inflammatory and autoimmune diseases. Whether it regulates the viral myocarditis (VMC) is unknown.

**Results:**

To examine the pathogenesis role of IL-23/Th17 axis in VMC, we used male BALB/c mice to induced VMC by Coxsackie virus B3 (CVB3) peritoneal injection. IL-23, IL-17, and signal transducer and activator of transcription 3 (STAT3) mRNA in the myocardium of VMC mice were assessed by semi-quantitative RT-PCR. IL-23 and IL-17 protein from blood serum were evaluated by ELISA. Phosphorylated-STAT3 (*p*-STAT3) protein expression in the myocardium was evaluated by immunohistochemical staining. Flow cytometric analysis was used to evaluate the frequencies of Th17 subsets. Isolated CD4^+ ^T cells from VMC mice were cultured with recombinant IL-23(rIL-23) *in vitro*. In addition, a STAT3-specific inhibitor (S3I-201) was used to test whether regulation of STAT3 could be partly responsible for Th17 diminution. Results showed that expression of IL-23, IL-17, STAT3 mRNA and protein increased in VMC mice. When purified CD4^+ ^T cells derived from VMC mice were cultured *in vitro *with rIL-23, the frequency of Th17 cells was dramatically increased, accompanied by significantly enhanced production of IL-17 in the supernatants of cultured CD4^+ ^T cells. S3I-201 significantly restrained Th17 cell proliferation.

**Conclusions:**

The IL-23/Th17 pathway axis is strongly expressed in murine VMC, identifying a novel pathway of potential significance in viral myocarditis.

## Background

Viral myocarditis (VMC) has been recognized as a cause of congestive heart failure for 50 years, but its diagnosis and treatment are still challenging. Coxsackie virus B3 (CVB3), a member of the Picornaviridae family, is the leading cause of VMC and often progresses to chronic myocarditis, dilated cardiomyopathy (DCM), and congestive heart failure, requiring heart transplantation [1]. Infection by CVB3 in a BALB/c murine model can induce myocarditis with a pathological process resembling human disease, and thus this model has been widely used for studying both the acute infectious phase and chronic immune phase of human VMC and cardiomyopathy [2]. Both the direct viral response and the excessive immune response (mediated especially by activated CD4^+ ^T lymphocytes) are the dominant causes of myocardial cell damage. In the acute stage of myocarditis, the production of autoantibodies mainly depends on the help of Th1 cells, which classical theories suggest are the main causative agent of myocarditis [3,4]. However, this concept has been challenged by the finding that mice lacking a typical Th1 cytokine (IFN-γ) are still susceptible to experimental autoimmune myocarditis (EAM) [5,6].

Two decades ago, Mosmann and Coffman proposed a model wherein T helper (Th) cells are divided into functional subsets on the basis of cytokine secretion, termed Th1 and Th2 [7]. IFN-γ and IL-12 initiate the differentiation of Th1 cells that are characterized by high production of IFN-γ and are indispensable for clearing intracellular pathogens. IL-4 triggers the differentiation of Th2 cells which are pivotal in organizing host defense against extracellular pathogens and in helping B cells to produce antibodies. Over the past several years, much progress has been made in understanding a distinct third Th17 lineage composed of IL-17-producing T-cells, Th17 cells are distinguished from the Th1 and Th2 cells not only by its unique profile of effector cytokines, but also in its development independently of key signaling elements that are central to Th1 or Th2 differentiation. This new CD4+ subset is important in providing protection against infection and maintaining chronic inflammatory diseases in autoimmune disease [8-11]. IL-23, a cytokine of the IL-12 family, is secreted as a heterodimer composed of a p40 subunit, identical to the p40 subunit of IL-12, and a unique p19 subunit, similar to the p35 subunit of IL-12. Recent results have shown that IL-23 is required for full acquisition of the pathogenic function and maintenance of effector Th17 cells [12-14]. Signal transducer and activator of transcription 3 (STAT3) signal transduction has emerged as a key component of Th17-dependent autoimmune processes, and STAT3 might affect the expression of IL-17 by increasing the expression of retinoid-related orphan receptor gamma t (RORγt) and retinoid-related orphan receptor alpha (RORα), which are upstream of IL-17 [15,16]. The IL-23/Th17 pathways are involved in the pathogenesis of rheumatoid arthritis, systemic lupus erythematosus, psoriasis, and other autoimmune diseases. These findings indicate the importance of the IL-23/Th17 axis in inflammatory and autoimmune responses. However, it is still largely unknown whether the IL-23/Th17 axis is involved in the regulation of myocardial inflammation. In this study, we examined whether the IL-23/Th17 cell axis is involved in myocarditis using a murine model of VMC induced by CVB3. We tested IL-23, IL-17, and Th17-specific transcription factor STAT3 mRNA expression in the myocardial tissue of VMC mice, IL-23 and IL-17 protein expression in serum, and phosphorylated-STAT3 (*p*-STAT3) expression in myocardium. We also evaluated the frequencies of Th17 subsets in spleen CD4^+ ^T cells. Finally, we assessed the possible modulating effect of recombinant IL-23 (rIL-23) and a STAT3-specific inhibitor (S3I-201) on Th17 cells *in vitro*.

## Materials and methods

### Animals

Specific pathogen-free inbred male BALB/c mice (6-weeks old) were purchased from Shanghai Laboratory Animal Center, Chinese Academy of Sciences, Shanghai, China (Certificate No.0062353 -SCXK (SH) 2007-0005). All animals were housed in a pathogen-free mouse room in an experimental animal center (Guangxi Medical University).

### Ethics Approval

The protocol was approved by the Committee on the Ethics of Animal Experiments of the Guangxi Medical University (ID: 20090622). All surgery was performed under sodium pentobarbital anesthesia, and all efforts were made to minimize suffering.

### CVB3 Titration in Hep-2 Cells

Heart-passaged CVB3 (Nancy strain, from Institute of immunology of Guangxi Medical University) was propagated in Hep-2 cells, cultured in monolayer and stored in a -80°C freezer. The supernatant from infected cell cultures was collected, and viral titers were determined in 96-well plates by the end-point dilution method. Briefly, 10-fold serial dilutions (1:10 to 1:10^-10^) of phosphate-buffered solution (PBS, Solarbio Science & Technology, Beijing, China) were prepared, and the 50% tissue culture infectious dose (TCID_50_) titer was determined by the cytopathic effects visible after 72 h. The TCID_50 _assay result for Hep-2 cells was 1 × 10^-7^.

### Myocarditis

CVB3 was diluted in PBS, and BALB/c mice were infected by an intraperitoneal (i.p.) injection of 0.1 ml of PBS containing approximately 100 TCID_50 _of the virus (n = 48) and randomly separated into 6 subgroups. Mice inoculated i.p. with PBS were taken as control (n = 30) and separated into 6 subgroups as well. The day when mice were injected i.p was defined as week 0, and myocardial tissues were harvested 0, 1, 2, 3, 4, and 6 weeks after i.p. injection. Hearts were cut longitudinally, fixed in 10% phosphate-buffered formalin, and embedded in paraffin. The myocardial tissues were cut into 5-μm sections at various depths in the heart and stained with hematoxylin & eosin to determine the level of inflammation, and graded in a blinded manner by a pathologist based on the following semi-quantitative scale: 0, no inflammatory infiltrates; 1, small foci of inflammatory cells between myocytes or inflammatory cells surrounding individual myocytes; 2, larger foci of 100 inflammatory cells or involving at least 30 myocytes; 3, 10% of a myocardial cross-section involved; and 4, 30% of a myocardial cross-section involved [17].

### Immunohistochemistry

Immunohistochemical staining was performed by the streptavidin-biotin complex method. Rabbit polyclonal antibodies against mouse IL-17 (Santa Cruz Biotechnology, USA) and *p*-STAT3 (Bioworld Technology, USA) at a 1:100 dilution were used as primary antibodies. The sections were washed and stained using the streptavidin-biotin complex kit (Boster, Wuhan, China) according to the manufacturer's manual. The procedures were as follows: After the sections were rehydrated, endogenous peroxidase activity was blocked with 3% hydrogen peroxide for 10 min at room temperature. After washing with distilled water, the sections were first incubated in 5% bovine serum albumin for 20 minutes at room temperature and then in the primary antibody for 24 h at 4°C. Next, the sections were washed with PBS and then incubated with the streptavidin-biotin complex for 20 minutes. Finally, the sections were developed with 3, 3-diaminobenzidine (Boster, Wuhan, China) and observed under a light microscope (Eclipse E800, Nikon, Japan). Non-immune goat serum was used instead of primary antibody as a control. IL-17 and *p-*STAT3 deposition in the cytoplasm and cytomembrane of myocardium were categorized semi-quantitatively according to the extent and intensity of staining using Image-Pro Plus Version 6.0 (Media Cybernetics, Bethesda, MD). Two pathology experts randomly selected 5 fields from each slice for blinded scoring and analysis by integrated optical density (IOD).

### Lymphocyte preparation

Spleens from virus-infected mice and controls were harvested aseptically. The lymphocyte fractions of these samples were obtained by Ficoll-Plaque (Solarbio Science & Technology, China) gradient centrifugation. Lymphocytes were maintained in a 24-well flat-bottom tissue culture plate with RPMI 1640 supplemented with 10% fetal calf serum (Gibco, USA) at 37°C in a humidified atmosphere with 5% CO_2_.

### Intracellular cytokine flow cytometry

Cytokine-producing cells were determined by intracellular staining using phycoerythrin-conjugated anti-mouse IL-17 (IL-17-PE) and phycoerythrin cyanine-5-conjugated anti-mouse CD4 (CD4-PE-Cy). Briefly, cells were stimulated with phorbol myristate acetate (PMA, 25 ng/ml, Sigma-Aldrich, USA), ionomycin (1 μg/ml, Sigma-Aldrich USA), and GolgiPlug (1 μl/10^6 ^cells, BD Biosciences) for 4 h. Cells were fixed in 4% paraformaldehyde, permeabilized with 0.1% saponin, stained with fluorescent antibodies against IL-17 and CD4, and analyzed on a FACSCalibur flow cytometer (BD Biosciences). CellQuest software (BD Biosciences) was used for data acquisition.

### CD4 ^+ ^T cell cultured and rIL-23 stimulation

Spleen lymphocytes were isolated from mice 1 week after CVB3 injection, and CD4^+ ^T cells were purified using a CD4^+ ^T cell isolation kit (Miltenyi Biotec MACS, Bergisch Gladbach, Germany). For culture, 10^6 ^CD4^+ ^T cells were activated for 5 d with 10 ng/ml of phytohemagglutinin (PHA) or 10 ng/ml of PHA + 10 ng/ml recombinant IL-23 (rIL-23, R&D). Cultured cells were restimulated with PMA/ionomycin in the presence of GolgiPlug for intracellular cytokine staining on day 6.

### CD4 ^+^T cell cultured with STAT3-specific inhibitor

Purified CD4^+ ^T cells (10^6^/ml) were cultured in complete culture medium with 10 ng/ml of PHA + 10 ng/ml recombinant IL-23 (rIL-23, R&D) with or without 100 μM of STAT3-specific inhibitor S3I-201 (NSC 74859, Santa Cruz Biotechnology, CA). Concentrations of S3I-201 were determined according to previously reports [18] and our preliminary experiments by culturing cells in the presence of increasing serial dosages of S3I-201. After 48 h of culture, cultured cells were restimulated with PMA/ionomycin in the presence of GolgiPlug for intracellular cytokine staining or for IL-17 mRNA transcript detection.

### Semi-quantitative RT-PCR detection of IL-23, IL-17, and STAT3 mRNA

Total mRNA was extracted from homogenized heart tissues using TRIzol Reagent (Invitrogen, USA) and then used to synthesize cDNA with an RT Kit (Ferma, USA). Reverse-transcription PCR was performed with first-strand cDNA synthesized with 1 μg of total RNA and oligo d(T)16 primers according to the manufacturer's instructions. The primers for the RT-PCR assays for IL-23p19, IL-17A, and Th17-specific transcription factor STAT3 were designed by Primer Premier 5.0 (Table 1). Mouse β-actin, a reference gene, was used to normalize each sample and each gene. Prepared cDNA was used for PCR amplification with the above primers under the following conditions: pre-heating at 94°C for 3 min, denaturing at 94°C for 30 sec, annealing at 64.3°C (IL-23), 64.9°C (IL-17) or 56.8°C (STAT3) for 30 sec, and extension at 72°C for 60 sec. The reaction repeated for 35 cycles followed by incubation at 72°C for 10 min. PCR products were analyzed by electrophoresis on a 2% agarose gel containing 0.5 mg/ml ethidium bromide. The resulting bands were observed and photographed under ultraviolet light and measured using the Digital Gel Imaging Analyst (Nikon 990-Doc 1000, USA). Density was determined for each sample PCR product, including the positive control. Background density was subtracted from each band, and the relative values of IL-23, IL-17, and STAT3 mRNA were calculated using β-actin mRNA as a standard. PCR products was sequenced by Songon Biotech Co., Ltd (Shanghai, China), and blasted in the NCBI Blast bank.

**Table 1:** Sequences of primers for RT-PCR

Molecule	Sequence (5' -3')	length
IL-23 [GenBank: 83430]	sense: 5'CTTCTCCGTTCCAAGATCCTTC 3' antisense:5'ACGCACTAGGTTTGCCGAGTAGA3'	200 bp
IL-17[GenBank:16171]	sense:5'GTCAATGCGGAGGGAAAG3' antisense: 5'CACGAAGCAGTTTGGGAC 3'	349 bp
STAT3 [GenBank: 20848]	sense: 5'CCCATATCGTCTGAAACTC3' antisense: 5'TTGCTCCCTTCTGCTCT3'	188 bp
β-actin [GenBank:11461]	sense:5'CCAGCCTTCCTTCTTGGGTAT3' antisense: 5'TTGGCATAGAGGTCTTTACGG 3'	102 bp

### Cytokine assay

Blood was collected via retro-orbital bleeding, and serum was separated. The amounts of IL-23 and IL-17 in the serum and culture supernatants were detected using the Quantified Mouse IL-23/IL-17 Immunoassay (R&D Systems, Minneapolis, MN). The limits of detection were 2.28 and 5 pg/ml.

### Statistical analysis

All data were expressed as mean ± SD. One-way ANOVA and the *q *test were used for comparison among groups. All data were analyzed with SPSS 16.0 for Windows. A significant difference was defined as *P *< 0.05.

## Results

### Development of VMC

**Figure 1:**
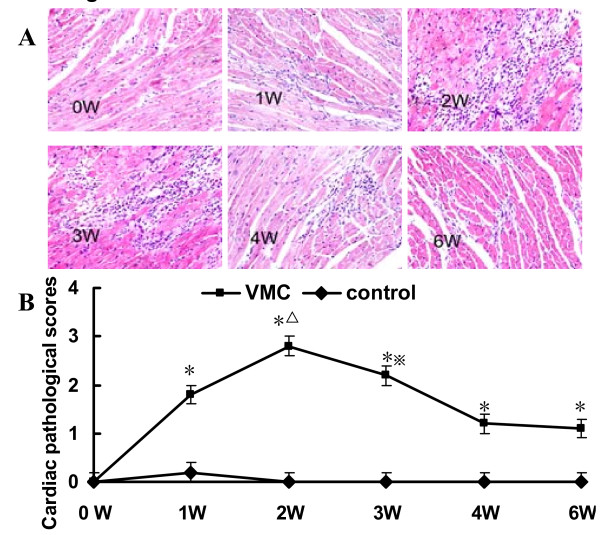
**Histopathologic changes of heart tissue in VMC mice**. A. Representative image of inflammation in VMC heart tissue (H&E, original magnification × 400). B The pathological scores at different times, *P < 0.05, versus control group, ^Δ^*P *< 0.05, versus week 0, 1, 3, 4, and 6 VMC mice, ^※^*P *< 0.05, versus week 4 and 6 VMC mice. The number of mice in each time point of VMC group was 8, 8, 5, 6, 6 and 5 respectively. The number of mice in each time points of control was 5.

CVB3-inoculated mice developed myocarditis one week after CVB3 infection, exhibiting with the symptoms of illness, including lethargy, progressive weight loss, and even death. The mice in control group remained healthy throughout the study. Histopathological observations of heart tissues were characterized by focal cellular infiltration with little necrosis or fibrosis, whereas no changes were seen in the controls [Figure 1A]. The pathological scores of the heart sections in the VMC group were significantly elevated compared with those in the control group [Figure 1B] and were accompanied by progressive cardiac inflammatory lesions that peaked at the second week. There were 8, 8, 5, 6, 6 and 5 mice remaining alive in each VMC subgroup. No mice died in control group.

### IL-23, IL-17 mRNA and protein expression were significantly elevated in the myocardium of VMC mice

**Figure 2:**
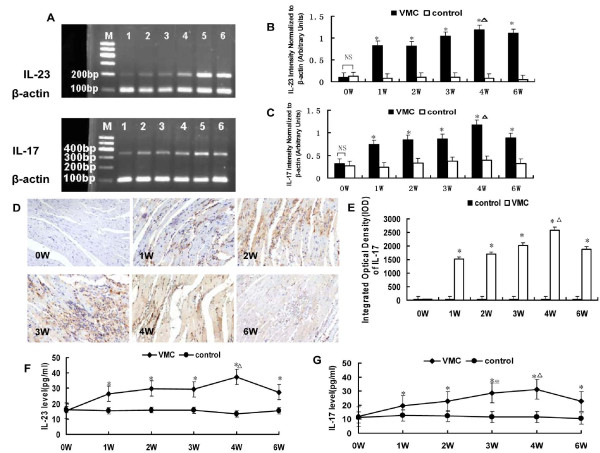
**IL-23, IL-17 mRNA and protein expression in the myocardium of mice**. A. Strong expression of IL-23, IL-17 occurred from the first week to the sixth week after CVB3 infection inVMC mice. M: Maker; 1: week 0; 2: week 1; 3: week 2; 4: week 3; 5: week 4; 6: week 6. B. Densitometric quantitation of IL-23. IL-23 mRNA expression was elevated in VMC mice from the first week to the sixth week. ^Δ^*P *< 0.01, versus control group, ^Δ^*P *< 0.05, versus week 0, 1, and 2 VMC mice. C. Densitometric quantitation of IL-17. IL-17 mRNA expression was elevated in VMC mice from the first week, and the arbitrary units were higher than those of controls. ^Δ^*P *< 0.01 versus controls, ^Δ^*P *< 0.05, versus week 0 VMC mice. D. Representative of IL-17 immunohistochemistry images in heart tissue of VMC mice (Dark brown granules, original magnification × 400). E. Morphometric quantitation of IL-17 protein expression. ^Δ^*P *< 0.01, versus control group, ^Δ^*P *< 0.05, versus week 0, 1, 2 and 6 VMC mice. F. IL-23 protein levels were steadily increased in the VMC mice, ^Δ^*P *< 0.05, versus control group, ^Δ^*P *< 0.05, versus week 0, and 6 VMC mice. G, IL-17 protein levels were steadily increased in the VMC mice from 1 week after i.p, and reached statistical difference comparing with those of controls, ^Δ^*P *< 0.05. The highest level of IL-17 in VMC occurred on 4th week, ^Δ^*P *< 0.05, versus week 0, 1 VMC mice. ^※^*P *< 0.05, versus week 0, 1 VMC mice.

Results showed that IL-23 and IL-17 transcripts were abundant in all samples from the first to the sixth week in VMC mice [Figure 2A]. The highest level of these two genes occurred on fourth week, while the expression of these two genes in controls remained constant throughout the procedure [Figure 2B, C]. IL-17 deposition in the cytoplasm and cytomembrane were positive in VMC mice 1-6 weeks after infection, which were statistical higher than those in control mice, *P *< 0.05 [Figure 2D, E]. The integrated optical density (IOD) of IL-17 protein in the myocardium of VMC and control mice at different time points were 35.98 ± 4.78 vs. 36.04 ± 5.46, 1516.41 ± 83.32 vs. 35.56 ± 4.51, 1700.95 ± 90.56 vs. 37.91 ± 6.70, 2013.95 ± 104.89 vs. 34.29 ± 8.43, 2570.12 ± 118.92 vs. 38.12 ± 9.52, and 1878.95 ± 90.89 vs. 35.67 ± 7.69, respectively. IL-23 protein levels increased in the serum of VMC mice in the first week [Figure 2F], peaked on the fourth week, and maintained high level till sixth week. The serum IL-23 protein levels at different times during VMC were 15.39 ± 6.14, 26.48 ± 5.98, 29.93 ± 12.05, 29.40 ± 11.73, 37.44 ± 8.51, and 27.56 ± 6.16 pg/ml. IL-17 protein levels increased in the serum of VMC mice from the first to the sixth week [Figure 2G]. After reaching the highest level on the fourth week, IL-17 sustained a high level till sixth week. The serum IL-17 proteins levels at different times during VMC were 11.88 ± 1.83, 19.64 ± 6.88, 22.70 ± 9.18, 28.77 ± 2.96, 31.27 ± 9.80, and 22.67 ± 4.47 pg/ml.

### Th17 frequencies were increased in the spleen one week after viral infection

**Figure 3:**
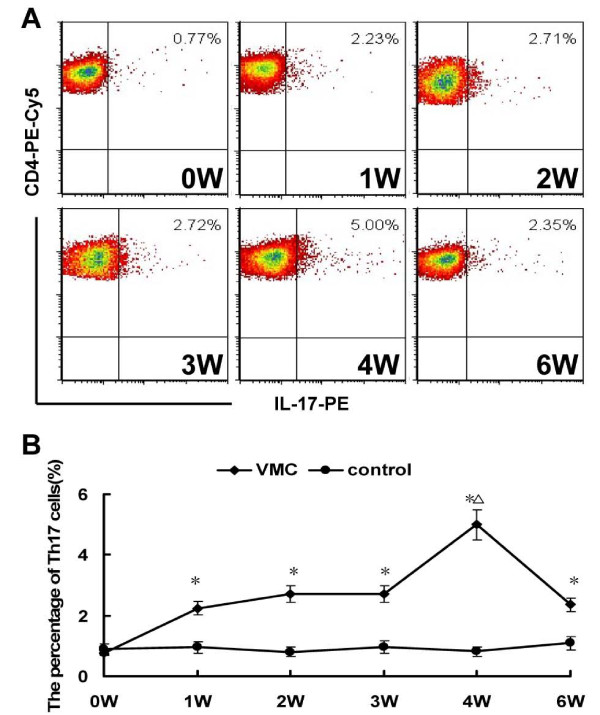
**Th17 frequencies in VMC mice**. A. Representative pictures of CD4^+^IL-17^+ ^Th17 cells in VMC mice. Numbers in the upper right quadrants indicate the mean percentages of CD4^+ ^Th17 cells in VMC mice. B. The results of the Th17 cells statistical analysis. ^Δ^*P *< 0.01, versus control group. ^Δ^*P *< 0.05, versus week 0, 1, 2, 3, and 6 VMC mice.

Compared to the controls, the frequencies of Th17 cells were markedly increased in VMC mice from the first week after viral infection [Figure 3A, B]. All of the results at different times were higher than those of controls except 0 week, *P *< 0.05. Th17 frequencies in each subgroup of VMC and control mice were 0.77 ± 0.32% vs. 0.89 ± 0.47%, 2.23 ± 0.89% vs. 0.95 ± 0.15%, 2.71 ± 0.82% vs. 0.80 ± 0.07%, 2.72 ± 0.64% vs. 0.96 ± 0.47%, 5.00 ± 0.81% vs. 0.98 ± 0.47%, and 2.35 ± 0.35% vs. 1.08 ± 0.66% respectively.

### STAT3 mRNA and p-STAT3 were high expressed in myocardium of VMC mice

**Figure 4:**
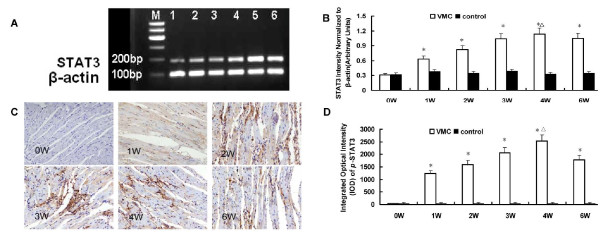
**STAT3 mRNA and *p*-STAT3 protein expression in the myocardium of VMC mice**. A. Strong expression of STAT3 occurred from 1st week to 6th week after CVB3 infection. M: Maker; 1: week 0; 2: week 1; 3: week 2; 4: week 3; 5: week 4; 6: week 6. B. Densitometric quantitation of STAT3. STAT3 mRNA expression was elevated in VMC mice from 1st week to 6th week, ^Δ^*P *< 0.01, versus control group. ^Δ^*P *< 0.05, versus week 0, 1 and 2. C. Representative of *p*-STAT3 immunohistochemistry images in heart tissue of VMC mice (Dark brown stain in cytoplasm, original magnification × 400). D. Morphometric quantitation of *p*-STAT3 protein expression in heart. ^Δ^*P *< 0.05, versus control group, ^Δ^*P *< 0.05, versus week 0, 1, 2 VMC mice.

Results showed that the expression of the Th17-specific transcription factor STAT3 mRNA was clearly elevated from the first week to the sixth week [Figure 4A, B]. The expression of this gene in controls remained constant throughout the procedure. Immunohistochemistry showed that *p*-STAT3 protein expressed in myocardium of VMC mice 1-6 weeks after CVB3 infection [Figure 4C, D]. The IOD of *p*-STAT3 protein in the myocardium of VMC and control mice at different time points were 33.67 ± 14.56 vs. 30.04 ± 12.90, 1234.41 ± 110.45 vs. 31.56 ± 13.45, 1600.95 ± 124.37 vs. 30.91 ± 19.30, 2070.12 ± 139.20 vs. 32.29 ± 17.82, 2524.25 ± 123.64 vs. 31.12 ± 16.89, and 1784.95 ± 130.19 vs. 33.67 ± 15.67, respectively.

### Th17 frequencies were increased by rIL-23 stimulation

**Figure 5:**
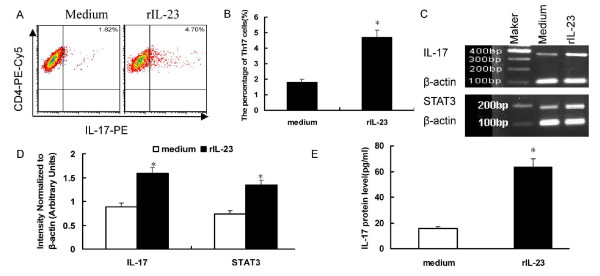
**Th17/IL-17 production stimulated by rIL-23**. A. Representative pictures of CD4^+^IL-17^+ ^Th17 cells stimulated by rIL-23. The numbers in the upper right quadrants represents the mean percentage of Th17 in response to 5 d of culture with or without IL-23. B. The results of Th17 cells statistical analysis. *P < 0.05. C. Strong expression of IL-17 and STAT3 after rIL-23 stimulation. D. Densitometric quantitation of IL-17 and STAT3 mRNA in the cultured cells after rIL-23 stimulation. *P < 0.05. E. IL-17 protein levels in the culture supernatant were significantly increased after rIL-23 stimulation *P < 0.01.

Those above results showed that IL-23/Th17 pathway is present in murine model of VMC. To confirm our *in vivo *data, we next cultured CD4^+ ^cells *in vitro *with rIL-23 to analyze cytokine production and Th17 cells. As our previous research had shown that Th17 cell increased one week after virus infection [17], we isolated CD4^+ ^T cells by MACS from BALB/c mice one week after CVB3 infection. The purity of CD4^+ ^T cell isolation was always >93%, as determined by flow cytometry. Cells were cultured in the presence of PHA (10 ng/ml) and rIL-23(10 ng/ml) or PHA (10 ng/ml) alone. After five days of culture, CD4-specific IL-17-producing Th17 cells were identified by FCS after PMA/ionomycin restimulation. Results showed that administration of rIL-23 promoted the expansion of Th17 cells (1.82 ± 0.17% vs. 4.70 ± 1.29%, *P *< 0.05) [Figure 5A, B], Corresponding, IL-17 and STAT3 mRNA expression in the cultured cells and IL-17 protein levels in the culture supernatants increased after rIL-23 stimulation [Figure 5C, D, E]. The IL-17 protein levels in the culture supernatants were 15.45 ± 5.67 vs.63.31 ± 12.94 pg/ml, respectively, *P *< 0.01.

### Th17 frequencies were decreased by treatment with S3I-201

**Figure 6:**
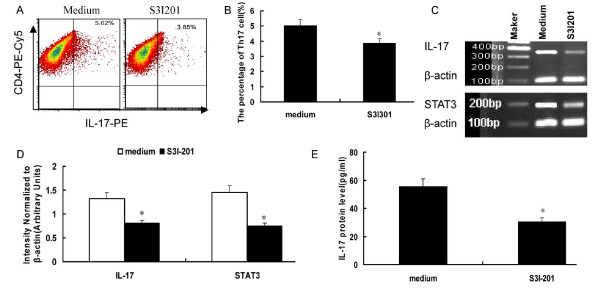
**Intracellular IL-17 production inhibited by S3I-201**. A. Representative pictures of CD4^+^IL-17^+ ^Th17 cells inhibited by S3I-201. The numbers in upper right quadrants represent the mean percentage of Th17 cells in response to S3I-201. B. The results of Th17 cells statistical analysis, *P < 0.05. C. Decreased expression of IL-17 and STAT3 after S3I-201 inhibition. D. Densitometric quantitation of IL-17 and STAT3 mRNA in the cultured cells after S3I-201 inhibition. *P < 0.05. E. IL-17 protein levels in the culture supernatant were significantly decreased after S3I-201 inhibition, *P < 0.01.

We used S3I-201, a STAT3-specific inhibitor, to test whether regulation of *p*-STAT3 could be partly responsible for IL-17 diminution. This agent selectively abrogates STAT3 DNA-binding activity *in vitro *by blocking the formation of STAT3:STAT3 dimers and is more than threefold more selective for STAT3 over STAT1. Results showed that administration of S3I-201 decreased the expansion of Th17 cells (5.02 ± 0.72% vs. 3.85 ± 0.69%, *P *< 0.05) [Figure 6A, B]. Correspondingly, IL-17 and STAT3 mRNA expression in the cultured cells and IL-17 protein levels in the culture supernatants decreased after S3I-201 inhibition [Figure 6C, D, E]. The IL-17 protein levels in the culture supernatants were 55.34 ± 10.78 vs. 30.28 ± 7.56 pg/ml, respectively, *P *< 0.01.

## Discussion

IL-12 mediated Th1 responses had been believed to increase CVB3-induced myocarditis in susceptible BALB/c mice, but results showed that IL-12 deiciency did not prevent the development of acute myocarditis[12], and mice lacking IFN-γ were highly susceptible to EAM [5,6], which means that these cells do not sustain or play decisive roles in myocardial autoimmune injury. At the same time, emerging data suggest that Th17 cells may play very important roles in VMC [19-21]. In addition, the IL-23/IL-17 axis, but not the IL-12/IFN-γ axis, is critical for the pathogenesis and development of certain autoimmune inflammatory diseases, such as inflammatory bowel disease and EAE [22-25]. However, whether IL-23/Th17 axis is involved in the pathogenesis of VMC is unknown.

This study observed the role of the IL-23/Th17 axis in VMC mice. Using semi-quantitative RT-PCR, we found that IL-23 and IL-17 mRNA expression was elevated in VMC mice immediately after CVB3 incubation, and the Th17-specific transcription factor STAT3 was robustly elevated compared to controls. IL-23, IL-17, and STAT3 protein levels were also higher in VMC mice than in controls. IL17-producing CD4^+ ^T cells were more abundant in the VMC mice. We next investigated whether the IL-23/Th17 pathway is important during a CVB3 challenge. We found that IL17-producing CD4^+ ^T cells were significantly higher in the presence of rIL-23 and accompanied by increased IL-17 transcription and protein levels, STAT3 specific inhibitor can decrease the frequency of Th17 cells. These results are consistent with papers suggesting that IL-23 is required for the development and expansion of Th17 cells [13,26]. The molecular mechanisms by which an adaptive immune response is skewed toward a Th17 response appear to rely in part on the ability of specific cytokines, specifically IL-23, that favor the development of Th17 cells. Activated macrophages and dendritic cells (DCs) secrete IL-23 in response to environmental danger signals. The receptor for IL-23 was described as being expressed in activated/memory T cell populations (but not in naive T cells) and specifically on Th17 cells. Furthermore, studies have proposed that IL-23R could be induced in a STAT3-dependent manner by TGF-β, IL-6, and IL-21[27,28], and IL-23R also appears to be dependent on RORγt [29]. Th17 cells require activation of STAT3 and subsequent RORγt induction. STAT3 activation is induced by IL-6, IL-21, and IL-23, and activated STAT3 directly binds to the STAT-binding sites on the IL-17 gene promoter, increases IL-17 gene transcription, and then affects the expression of IL-17 by increasing the expression of RORγt and RORα [15,16]. Therefore, STAT3 and RORγt seem to cooperate, and competent production of IL-17 depends on the presence of both transcription factors. The present study suggests that, upon stimulation with CVB3, pathogen-associated molecular patterns activate specific Toll-like receptors (TLRs) or dectin receptors, and DCs secrete IL-23 along with other cytokines such as IL-6, IL-21 and TGF-β. In addition, the combined signals of RORγt and STAT3 might promote IL-23R expression. When the IL-23 cytokine signal becomes dominant, it skews toward the type of immunity that develops Th17 cells and confers responsiveness to IL-23/Th17. As a result, IL-23 and IL-17 mRNA expression and protein secretion would be abnormally elevated.

Some limitations need to be acknowledged. First, like most cytokine receptors, IL-23R is expressed at low levels, and it is difficult to purify Th17 cells on the basis of IL-23R expression. An alternative selection sign is the chemokine receptor CCR6, which is reported to be a predominant marker of Th17 cells. CCR6 expression on CD4^+ ^T cells is able to distinguish Th17 cells from most other Th cells, with the possible exception of a subset of Treg [30]. However, CCR6-expressing Th17 and Treg cells may coexist in inflamed tissue in the context of an inflammatory condition [31,32]. As a result, we could not isolate a specific subpopulation of Th17 cells in VMC mice to culture *in vitro*. Second, besides being produced by Th17 cells, IL-17 is also produced by a variety of cell types, including γδT cells, NKT cells, NK cells, neutrophils, and eosinophils, and whether these IL-17-producing cells contribute to the pathogeneses of myocardium injury during VMC needs more research.

In summary, we have demonstrated that the IL-23/Th17 pathway is frequently induced in a murine model of VMC, and this pathway may therefore play a pathogenic role in VMC. However, the precise function of IL-23 in Th17 cells remains elusive, in part because the timing and consistency of IL-23R expression in T cells have been difficult to investigate. Furthermore, autoimmune diseases have so far been associated with Th1 and Th17 cells. Although the latest advances, together with ours, suggest that Th17 is a driving factor of disease pathogenesis in VMC, data suggested that Th1 cells can induce myocarditis with severity similar to Th17 cells [33], IL-17A knockout mice are not fully protected against EAM and still develop mild myocarditis [34]. In addition to these, results show that a severe autoimmune disease can be elicited in the absence of IFN-γ and IL-17, the prototypic Th1 and Th17 cytokines. Besides, because both IFN-γ^+ ^and IL-17^+ ^CD4^+ ^T cells can be detected in diseased mice[35,36], Is myocarditis a simple Th1 or Th17 driven disease, or there is considerable overlap between the developmental pathways that produce IFN-γ^+ ^and IL-17^+ ^effector cells? Probably, a model of disease pathogenesis in either Th17 cells or Th1 cells favor disease is too simplistic, comparative studies are required to further reveal the roles of these cytokines in the pathogenesis of immune-related diseases.

## Competing interests

The authors declare that they have no competing interests.

## Authors' contributions

YF participated in data collection, coordinated the study, performed the statistical analysis and interpretation of data, prepared the draft of manuscript and reviewed it. WW conceived of the study and designed it, coordinated the study and reviewed it. YY, KQ, PY carried out data collection. HY participated in design of the study and reviewed the manuscript. All the authors have read and approved the final manuscript.
